# Editorial: Neuroinflammation and Neuroautoimmunity in Peripheral Neuropathies: Old Players, New Roles

**DOI:** 10.3389/fimmu.2021.801760

**Published:** 2021-11-23

**Authors:** Sara Marinelli, Maria Maiarù, Alessandra Colciago

**Affiliations:** ^1^ Consiglio Nazionale delle Ricerche, Institute of Biochemistry and Cell Biology, Monterotondo, Italy; ^2^ Department of Pharmacology School of Pharmacy, University of Reading, Reading, United Kingdom; ^3^ Department of Pharmacological and Biomolecular Science, University of Milan, Milan, Italy

**Keywords:** peripheral neurodegeneration, neuropathic pain, neuroimmune crosstalk, glia, autoimme diseases, innate immunity, chronic and inflammatory pain, biomarkers

In the last two decades, great emphasis has been placed on neuroimmune interaction in neuropathy and neuropathic pain onset (1). The importance of the immune system and its mediators in orchestrating, cooperating, and/or supporting mechanisms triggering or facilitating peripheral painful and non-painful neuropathy has also been recognized. Despite growing and continuous interest (2) in this topic, the links and communication among all the players (immune cells, peripheral and central glia, neurons) are complex and new elements are still required to decipher the underlying processes in different pathological conditions.

Although peripheral neuropathies are of different aetiology (traumatic, metabolic, iatrogenic, autoimmune, genetic), they share similar peripheral and central neurodegenerative and neuroinflammatory processes. In particular, accumulating evidence highlights the critical role of crosstalk among neural, glial, and immune cells (3–5). Physiological and aberrant communication among different cell types takes place through the release of mediators having a pro- or anti-inflammatory power able to determine a cell conformational, morphological, and/or functional change (phenotypical switch); this, in turn, amplifies and supports the neuroinflammation or, on the contrary, switches off or reduces the neuropathy progression and symptoms such as neuropathic pain.

In light of this inclusive approach, in which all neuroimmune players are in a reciprocal relationship, the papers included in this Topic Research provide a multifaceted view of the topic, coming from different models of disease. The aim is to suggest new potential therapeutic targets to improve the quality of life of patients with these pathologies.

## Consolidation of Neuroimmune Crosstalk in Neuropathy Mechanisms (Old Players)

From an overall view of the published papers considering different neuropathy models including Chemotherapy-Induced Peripheral Neuropathy-CIPN (Fumagalli et al.), Monosodium IodoAcetate (Kwok et al.), Chronic Constriction Injury- CCI (Kwiatkowski et al.), and neurodegenerative diseases such as Amyotrophic Lateral Sclerosis (ALS) (Angelini et al.), or trauma-related injuries (Guillame-Barrè) (Huang et al.), findings emerge to indicate that immune cells and their mediators influence the glia and sensory neurons response. During the development of neuropathy, regardless of the original cause, Wallerian degeneration (WD) (such as in the case of traumatic peripheral nerve damage) or Wallerian-like degeneration (WlD) (such as in metabolic or immune-mediated neuropathies) occur, axonal degeneration and dysmyelinating phenomena take place, and neuropathic pain can arise.

Consequently, a step-by-step and well-established process begins evolving immediately after the lesion. Schwann cells can remove and clear myelin and axonal debris, and later (within one week from lesion) resident immune cells (innate immunity) in the nerve. In response to the insult, macrophages, leukocytes, mast cells, glial cells, and T lymphocytes are gradually involved in neurodegenerative progress, contributing, throughout the release of cytokines, chemokines, and trophic factors, to the “inflammatory soup” (6). Each of these mediators can be considered not only biomarkers, identifying a specific stage of the disease, but also “messages” utilized by the cells to communicate with each other. Moreover, they can be potential therapeutic targets.

## New Evidence: Biomarkers, Mechanisms, and Therapeutic Targets (New Roles)

Given the determining role of inflammation and its mediators in peripheral neuropathies, what is still lacking is the identification of early and precise diagnostic markers for different pathological conditions as well as the possibility to prevent, stop or slow down the progression of the diseases and to reduce the related pain symptoms. In this regard, some innovative views are suggested in this Research Topic. Diagnostic tools might take advantage of the analysis of specific long non-coding RNAs (lncRNAs) expression, which have been proposed as a sensitive diagnostic panel for acquired immune-mediated polyneuropathies (Hussen et al.) in which a precocious diagnosis might represent a crucial point for neurodegenerative diseases. The importance of an early diagnosis is also underlined in an interesting paper by Angelini et al. in which, for the first time, they reveal a WlD immune-mediated in ALS mice. The very initial peripheral nerve degeneration underpins early biomarkers (IL-6 and IL-10) as well as the involvement of innate immunity (mast cells and macrophages) in inflammatory phenomena and neurodegeneration, which is useful from a therapeutic perspective. Regarding immune-mediated neuropathy, an innovative perspective in the Guillame-Barré Syndrome aetiology is offered by the study by Huang et al. The authors suggest that GBS can be triggered not only by infection or vaccination but also by trauma, proposing the concept of “trauma-related GBS”, which is useful in understanding pathogenic mechanisms.

Most of the pro-inflammatory agents released during neuropathy development can facilitate and support the onset of chronic pain. Pro-inflammatory agents can be the cause of painful response and, given the inefficacy of common analgesic drugs and opioids, chemokine and cytokine modulation is a desirable aim in patient management (Fumagalli et al.).

Inflammatory agents are released both at the peripheral and spinal levels: microglia reactivity is critical for the initial phase of neuropathic pain, therefore targeting the mechanisms essential for its activation might prevent pain chronicization and astrocyte recruitment. The work of Kwok et al. demonstrates that injury in primary joint afferents in a model of arthritis activates the joint nociceptors, triggering a central spinal microglia response *via* ATP signaling (Kwok et al.).

Since the microglia plays the immune system role at the CNS, it is equipped to interplay with the majority of immunomodulators. Kwiatkowski et al. elegantly show, in a model of peripheral traumatic injury, that the simultaneous blockade of the CC chemokine receptors type 2 (CCR2) and 5 (CCR5) with the dual CCR2/CCR5 antagonist, cenicriviroc, reduces microglial activation and pain-related behaviors and potentiates the efficacy of opioids.

Other similar confirmations are outlined in an intriguing paper by Moschetti et al., in which inhibition of the Prokineticin 2 receptor with the antagonist PC1, in a model of CIPN, reduces or even prevents the release of cytokines (PK2, PK-R1, TLR4, IL-6, IL-10), impeding the neurotoxic effects of chemotherapeutics on primary sensory neurons.

## Conclusion

In the last few years a wide and comprehensive picture, detailing the participation of inflammatory/immune cells and mediators in peripheral neuropathies, has been progressively developed. Despite this, a number of actors and their specific mechanisms of action still need to be precisely positioned in this panel. When this complex figure is more precisely delineated, researchers will be able to achieve specific and valuable therapeutic interventions.

## Author Contributions

SM, MM, and AC equally contributed to the editorial.

## Conflict of Interest

The authors declare that the research was conducted in the absence of any commercial or financial relationships that could be construed as a potential conflict of interest.

## Publisher’s Note

All claims expressed in this article are solely those of the authors and do not necessarily represent those of their affiliated organizations, or those of the publisher, the editors and the reviewers. Any product that may be evaluated in this article, or claim that may be made by its manufacturer, is not guaranteed or endorsed by the publisher.
